# Supplementary figures and images for: Inference of B cell clonal families using heavy/light chain pairing information
Source: PLoS Comput Biol. 2022 Nov 28;18(11):e1010723. doi: 10.1371/journal.pcbi.1010723 (PMC9731466; doi:10.1371/journal.pcbi.1010723)

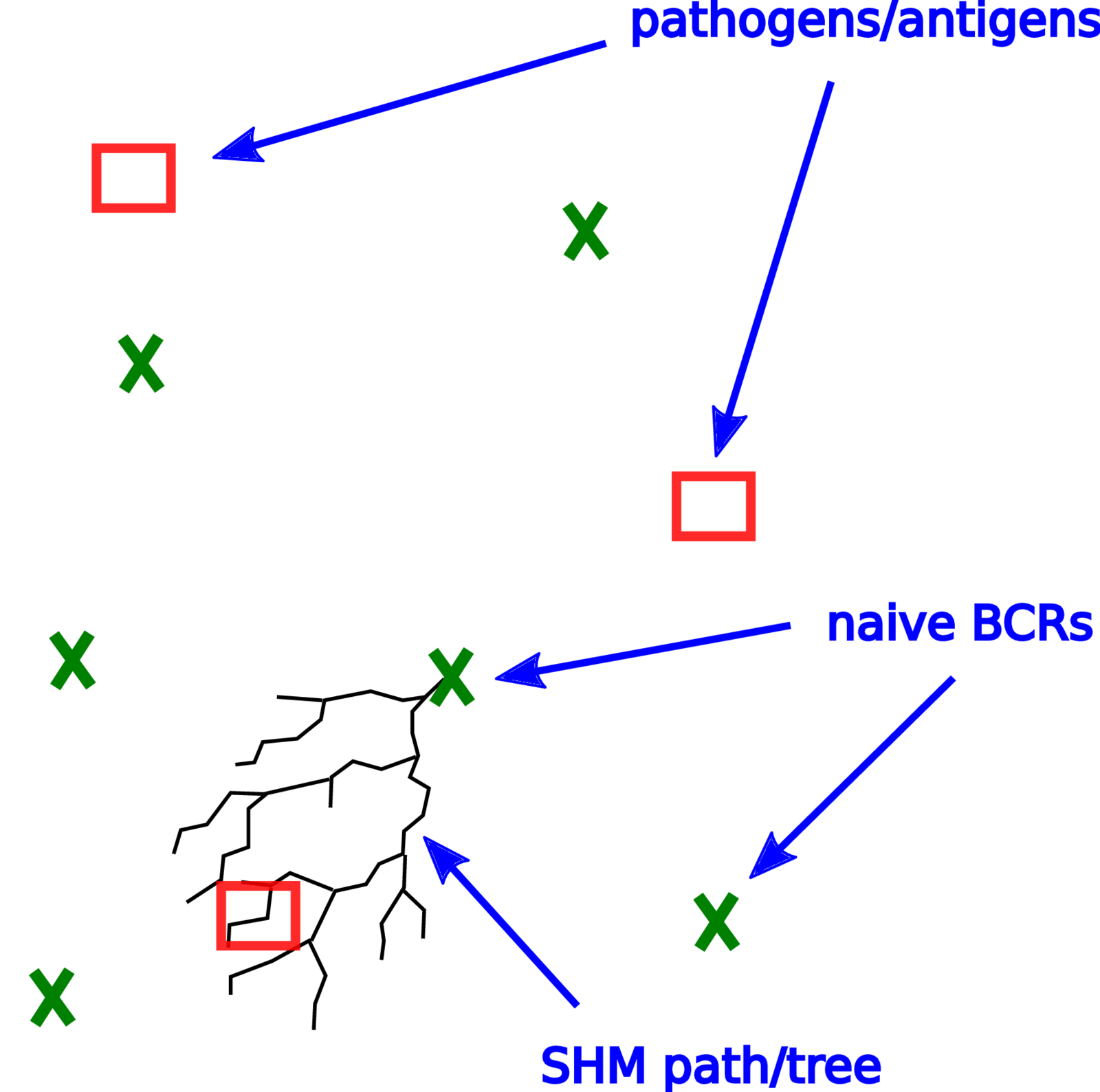

Supplement: S1 Fig — The immune system populates the space with naive rearrangements (green crosses) such that any antigen (red squares) will be somewhat near to an antibody. After stimulation by its cognate antigen, a B cell will migrate to a germinal center and undergo affinity maturation to move its offspring closer to the antigen (black tree). (TIFF) [file pcbi.1010723.s001.tiff]

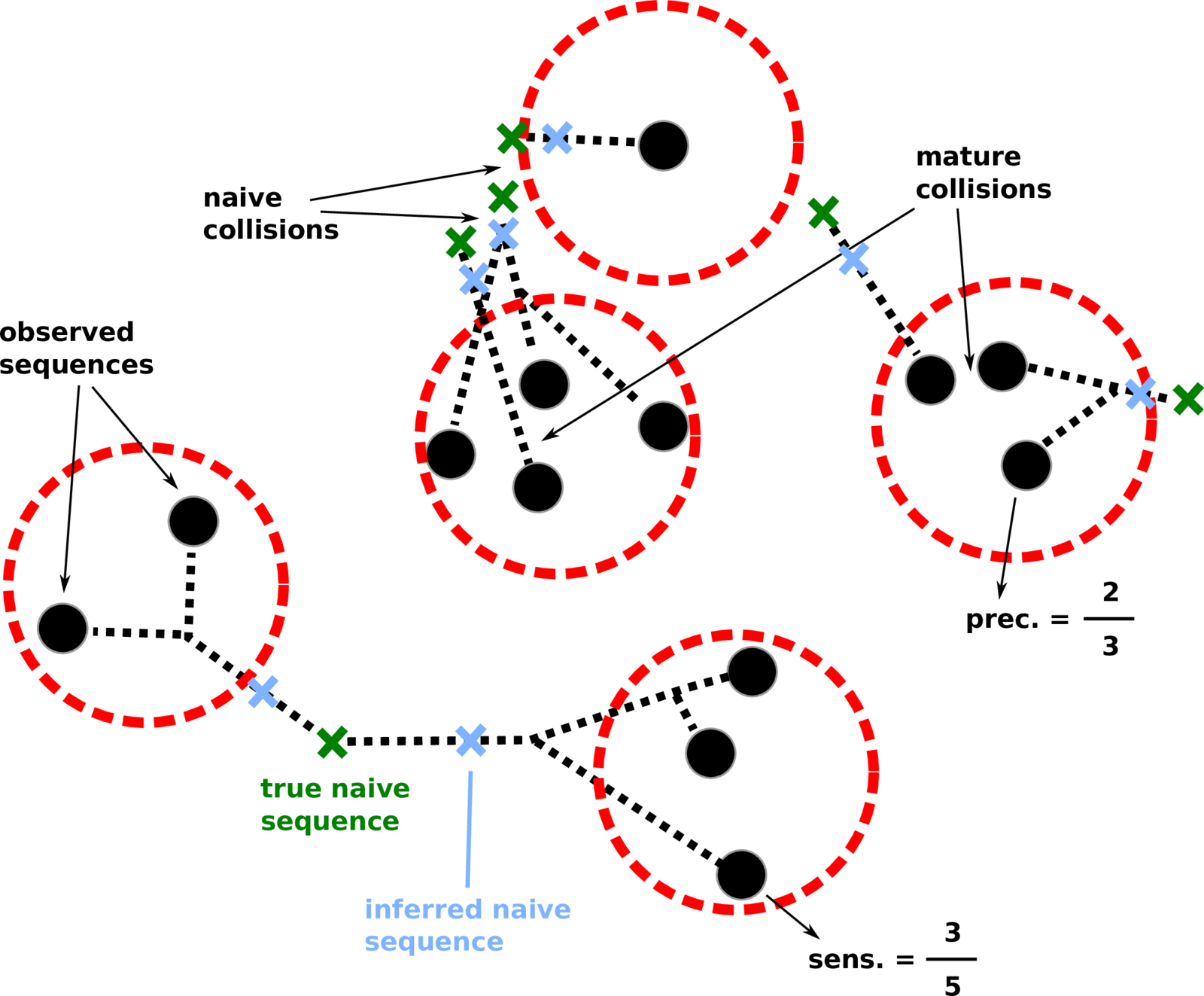

Supplement: S2 Fig — If clustering (red circles) is performed on observed (mutated) sequences (black dots), sequences in a family are unnecessarily far from each other. It is much better to cluster on the inferred naive ancestor (blue crosses) of each sequence (and, as clustering progresses, the naive ancestor inferred on the entire cluster at that point in time). Clustering on observed sequences thus conflates inferred SHM (distance from black dots to blue crosses) with inference inaccuracy (distance from blue crosses to green crosses). Note that this figure also shows a potential problem with using shared mutations as a criterion for clustering: while it can help in grouping together members of a sublineage in a high mutation environment, it assumes that all families consist of only a single sublineage, and will thus spuriously split families that do not (such as the five-sequence family at bottom left above). We also show examples of the precision and sensitivity calculation for one sequence each; these would then be averaged over all sequences to arrive at the values for the entire partition. (TIFF) [file pcbi.1010723.s002.tiff]

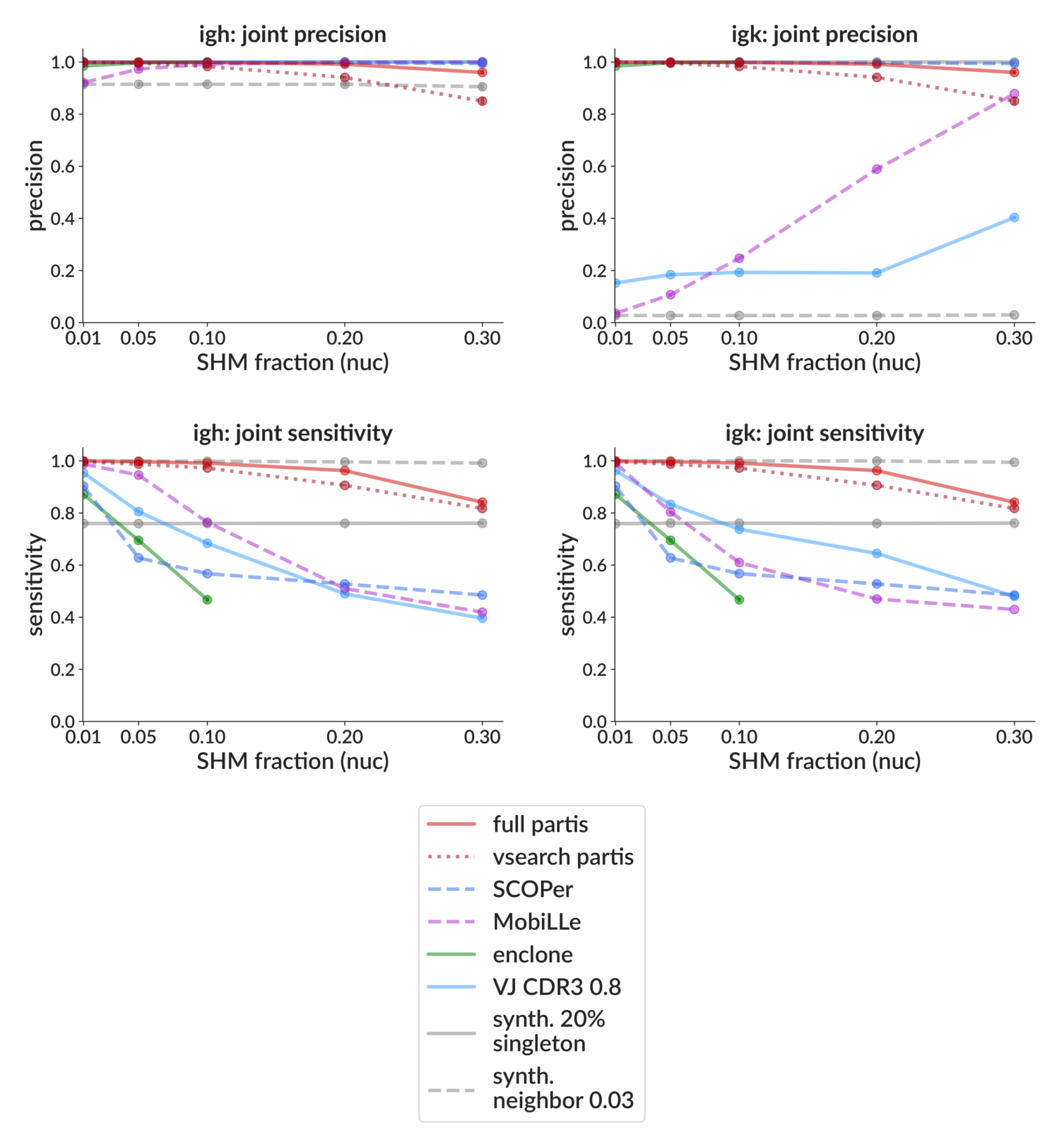

Supplement: S3 Fig — See Fig 3, which combines these values into the F1 score, for details. (TIFF) [file pcbi.1010723.s003.tiff]

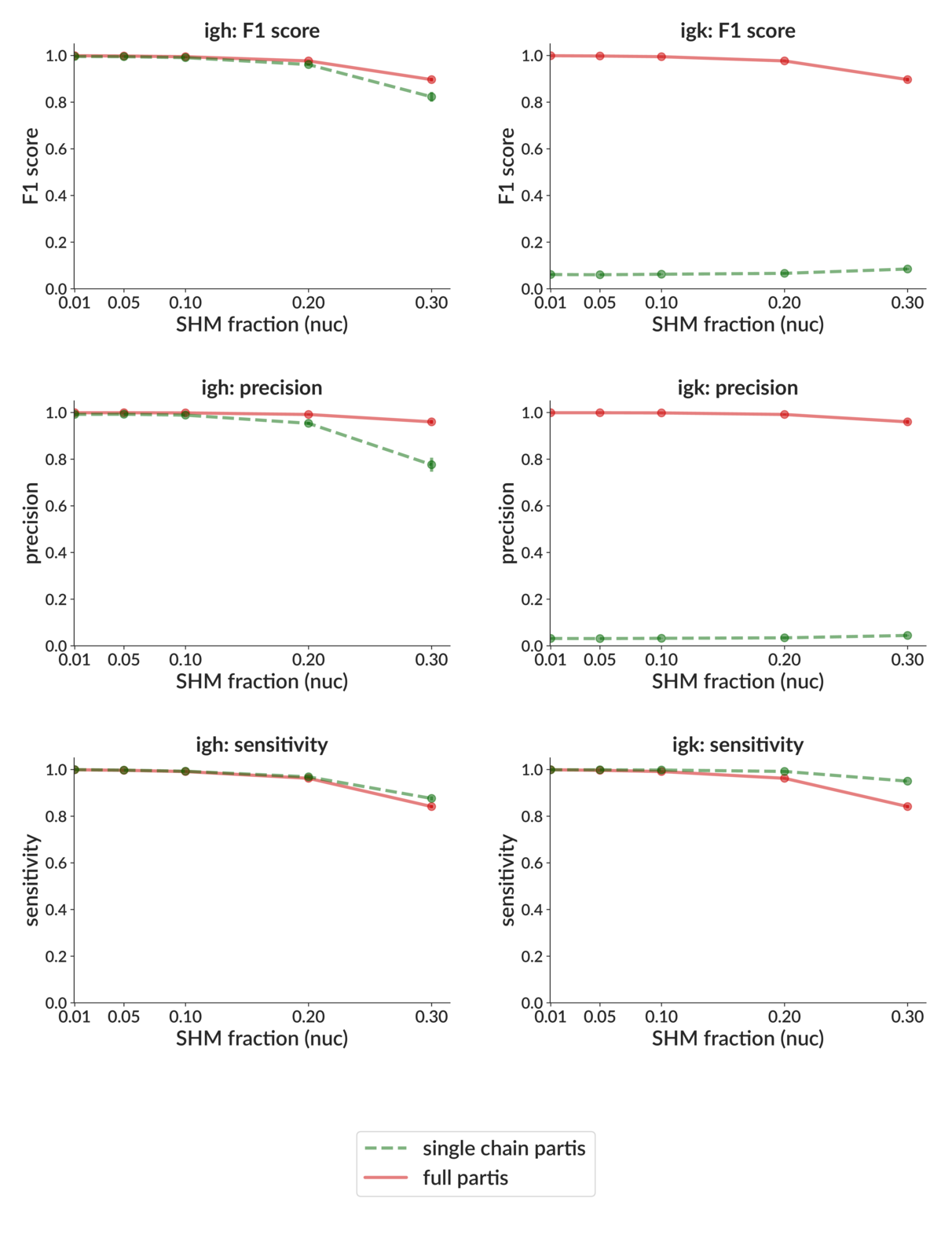

Supplement: S4 Fig — See Fig 3, for details and compare to S5 Fig for SCOPer. Shown also vs. number of families in S7 Fig. (TIFF) [file pcbi.1010723.s004.tiff]

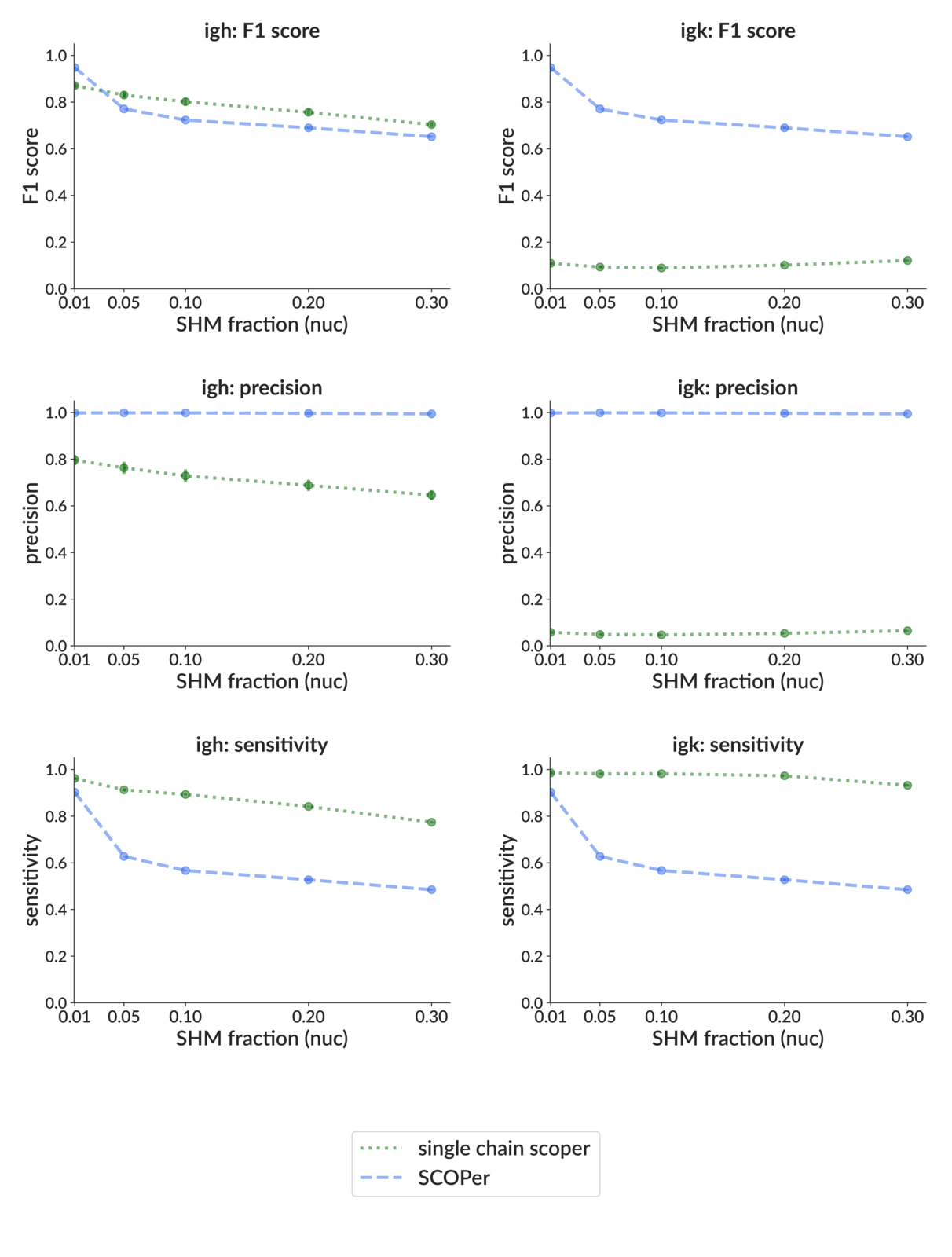

Supplement: S5 Fig — See Fig 3 for details and compare to S4 Fig. for partis. (TIFF) [file pcbi.1010723.s005.tiff]

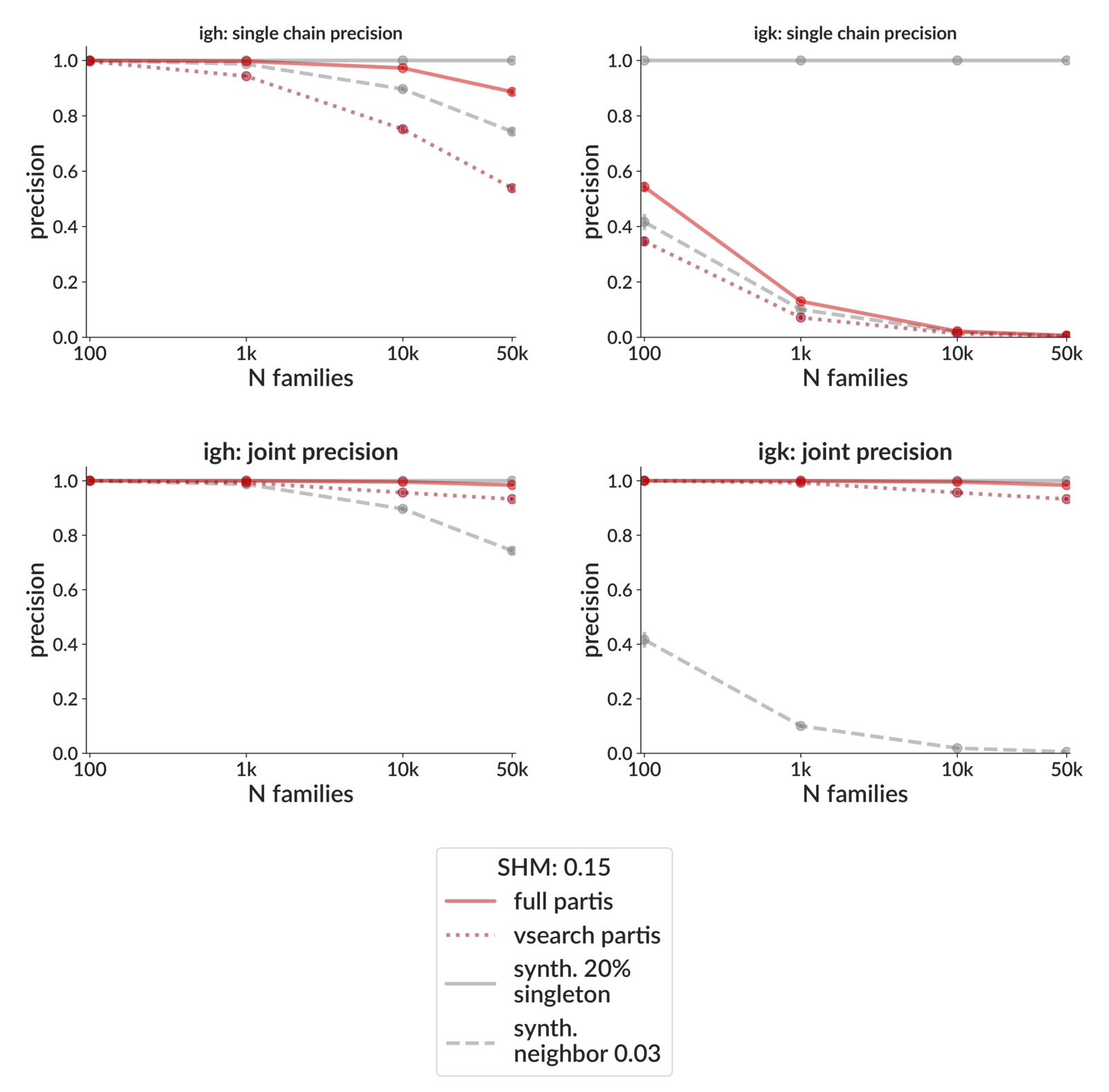

Supplement: S6 Fig — In order to focus on the effect of family collisions (unrelated families that are close to indistinguishable), we show performance only in terms of precision, and on samples consisting only of singletons. Each point is the mean (± standard error, often smaller than points) over three samples with 15% mean nucleotide SHM, each consisting of the indicated number of singleton families. See Fig 3 for details, and S7 Fig. to compare single vs. paired partis. (TIFF) [file pcbi.1010723.s006.tiff]

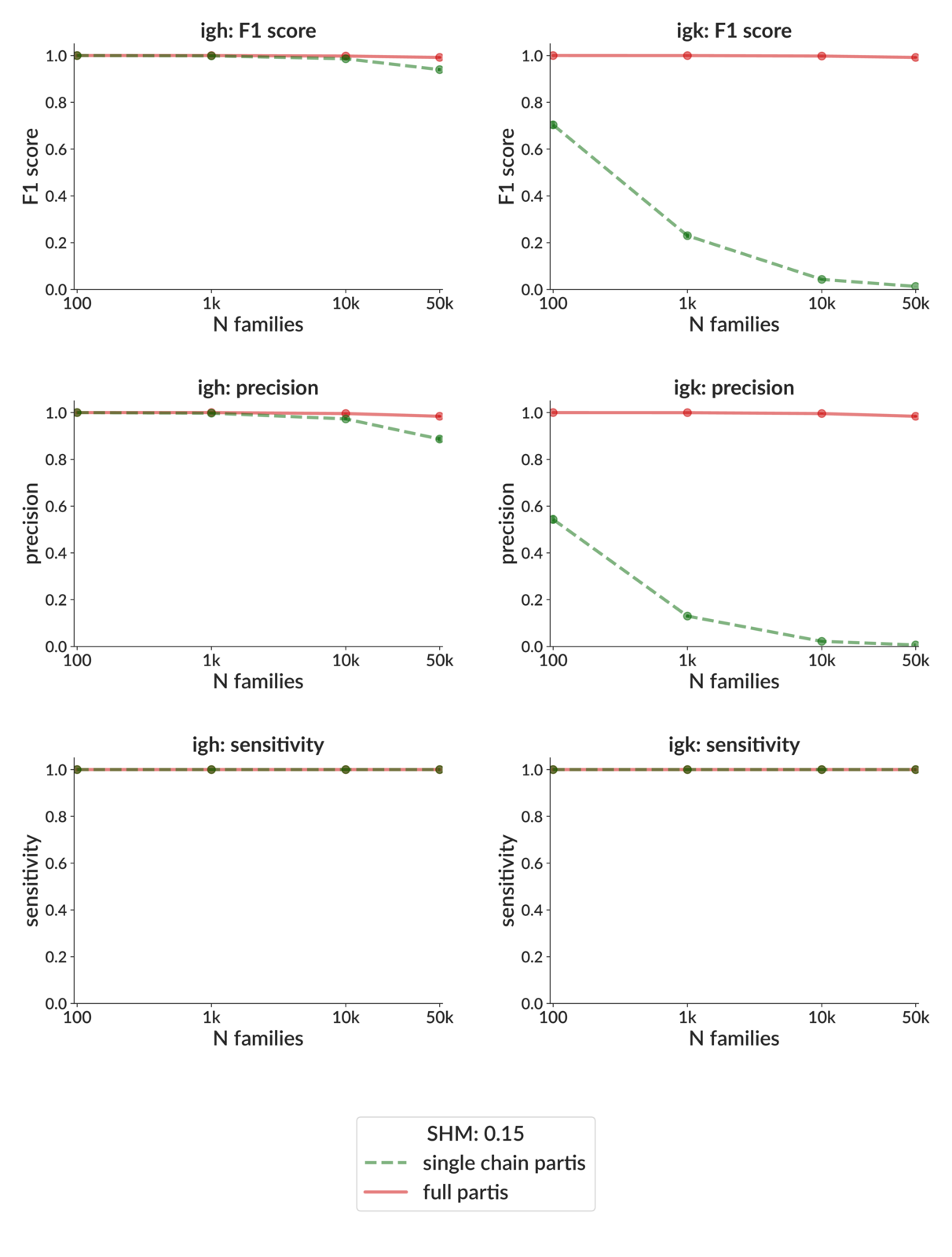

Supplement: S7 Fig — See S6 Fig for details, and S4 Fig for comparison vs. SHM. (TIFF) [file pcbi.1010723.s007.tiff]

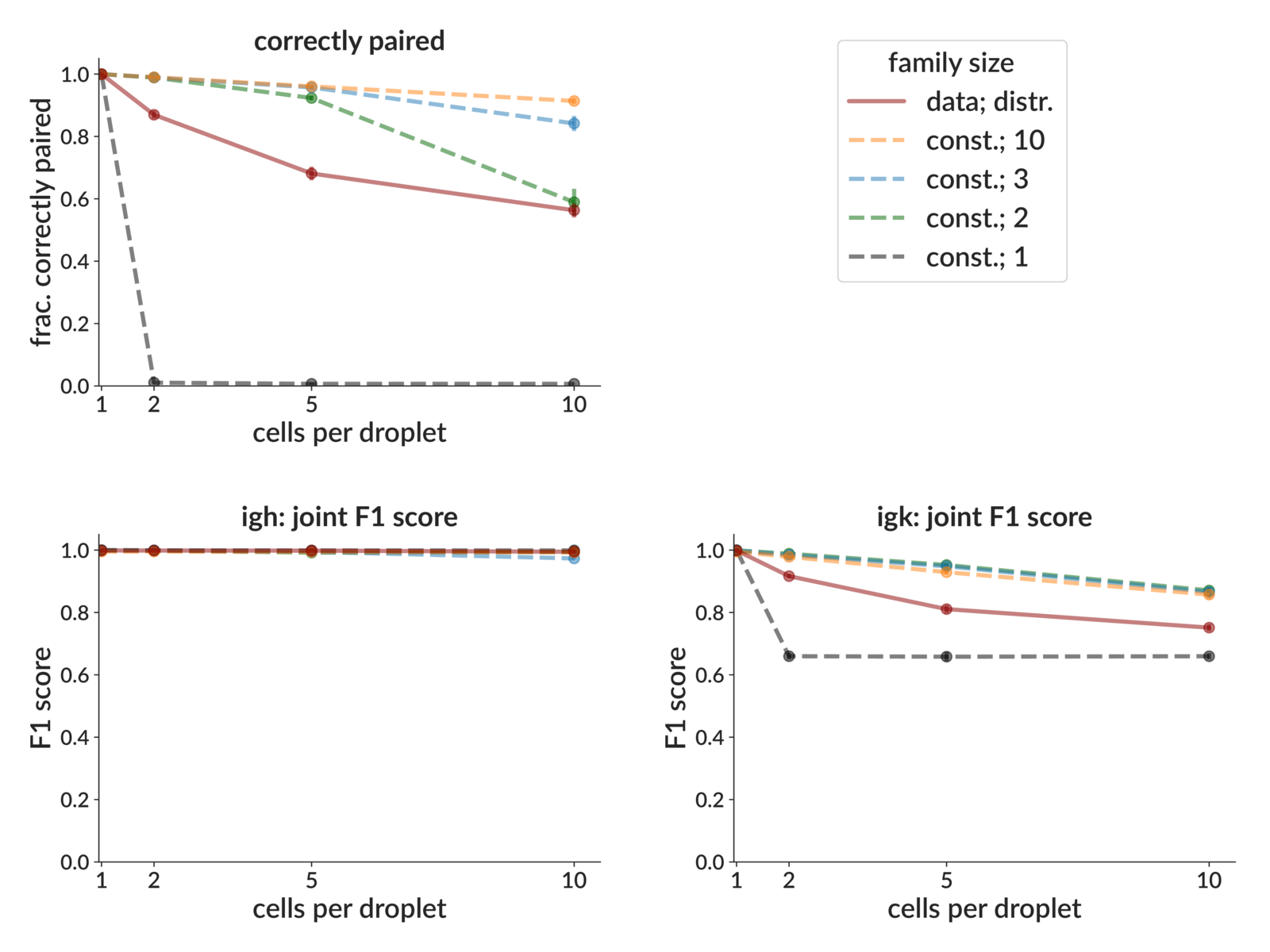

Supplement: S8 Fig — Results shown for samples with family sizes drawn from a distribution inferred from real data (solid red line; corresponds to Fig 4), and where all families have the same, indicated size (dashed lines). Each point is the mean (± standard error, often smaller than points) over three samples, each consisting of 3,000 simulated rearrangement events. With no pair info cleaning, any cells that share droplets (i.e. all points to the right of 1) would have no pair info, which results in performance as shown for single-chain clustering, with very poor IgK precision (S4 Fig middle right, dashed green). (TIFF) [file pcbi.1010723.s008.tiff]

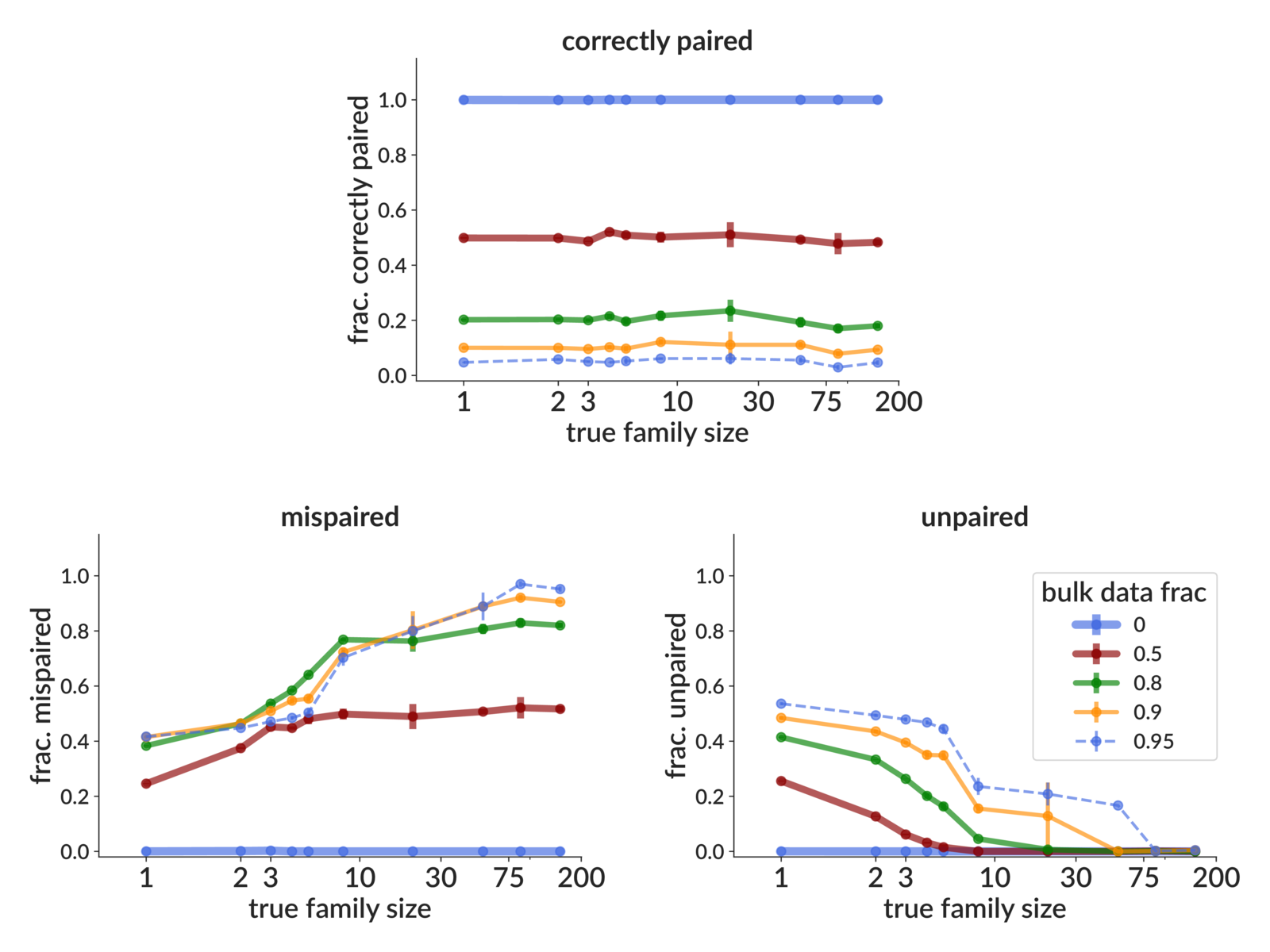

Supplement: S9 Fig — Note that essentially by construction, the fraction correctly paired is simply the paired (non-bulk) fraction of the sample; the goal of the method is to pair sequences with a sequence from the correct (or a similar) family, but not necessarily the correct sequence (Fig 5). (TIFF) [file pcbi.1010723.s009.tiff]

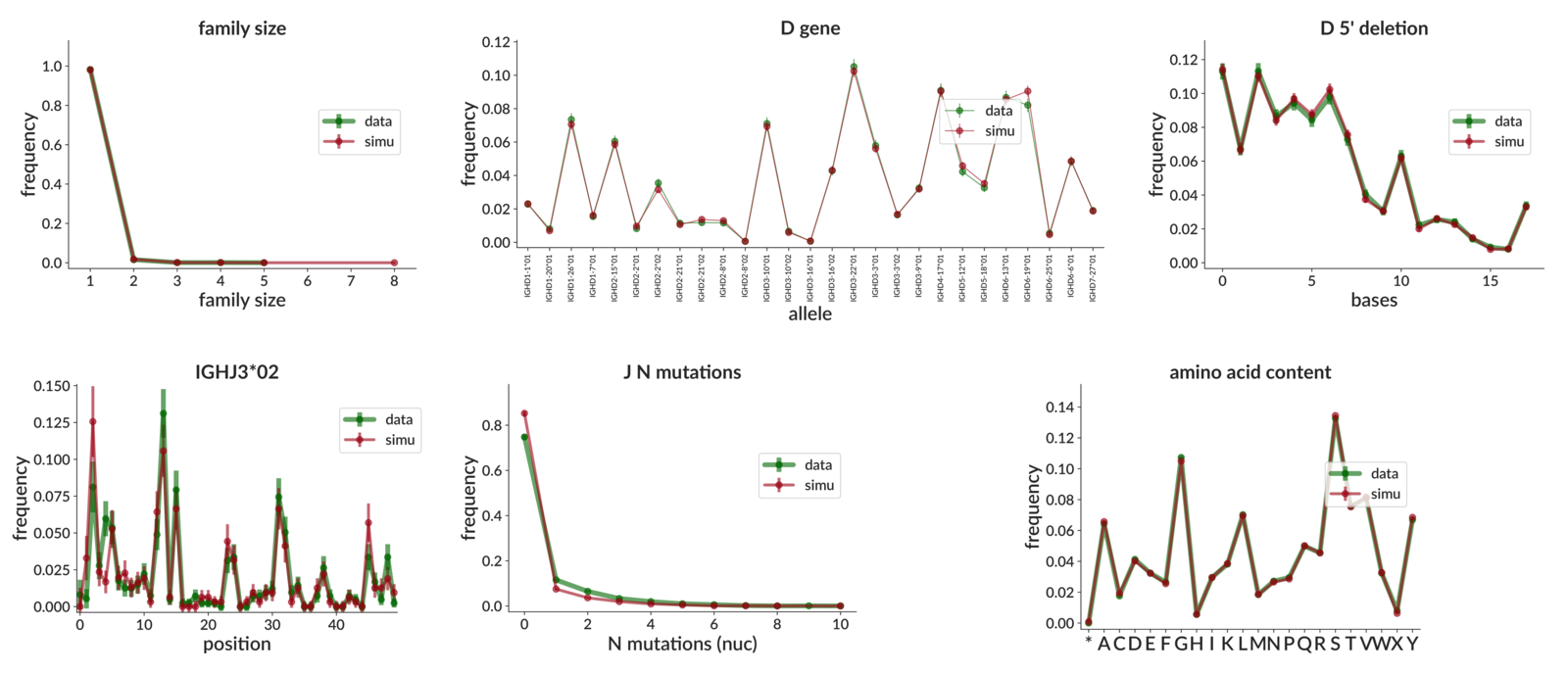

Supplement: S10 Fig — Shown here are cluster size distributions (top left), D gene usage (top middle), D 5’ deletion lengths for all D genes together (top right), per-position SHM frequencies for IGHJ3*02 (bottom left), number of J segment mutations (over all J genes, bottom middle), and sequence amino acid content (bottom right). Distributions for all other parameters, and for the same studies performed on three other real data samples, may be found at https://doi.org/10.5281/zenodo.5860143. (TIFF) [file pcbi.1010723.s010.tiff]

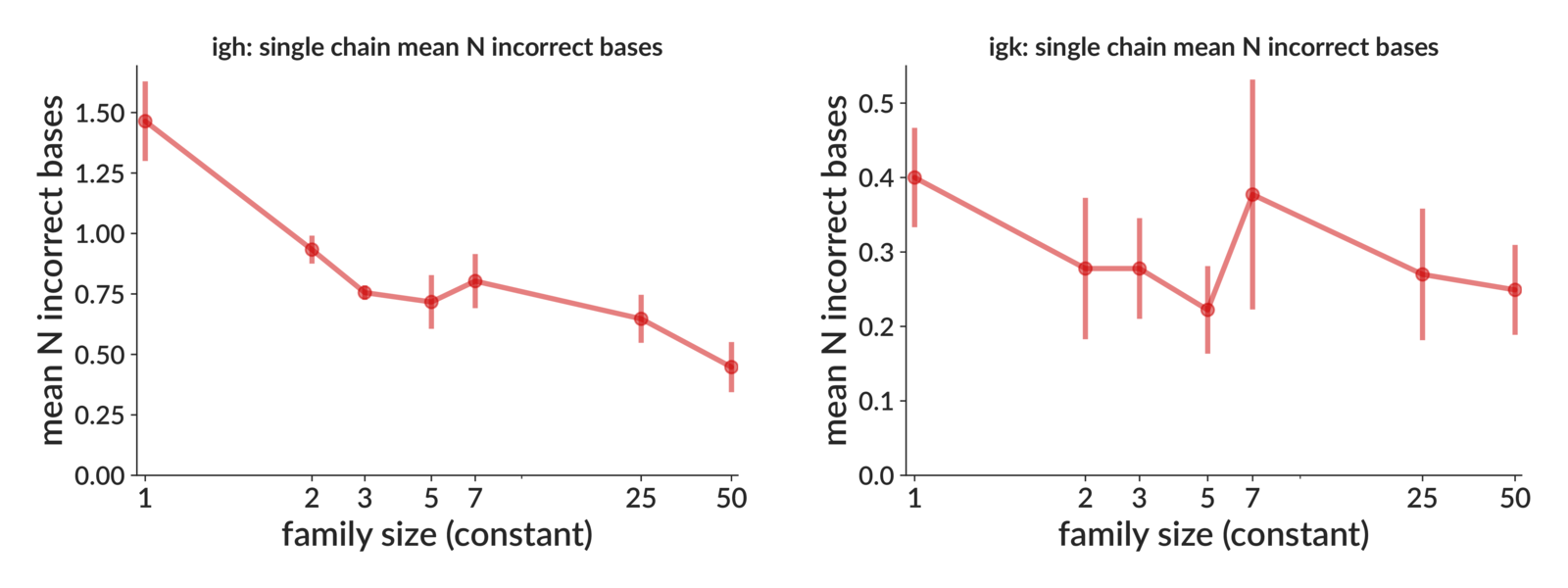

Supplement: S11 Fig — Accuracy is measured as the Hamming distance separating the true and inferred naive sequences. Each point is the mean (± standard error) over three samples, each consisting of 50 simulated rearrangement events with the indicated size. (TIFF) [file pcbi.1010723.s011.tiff]

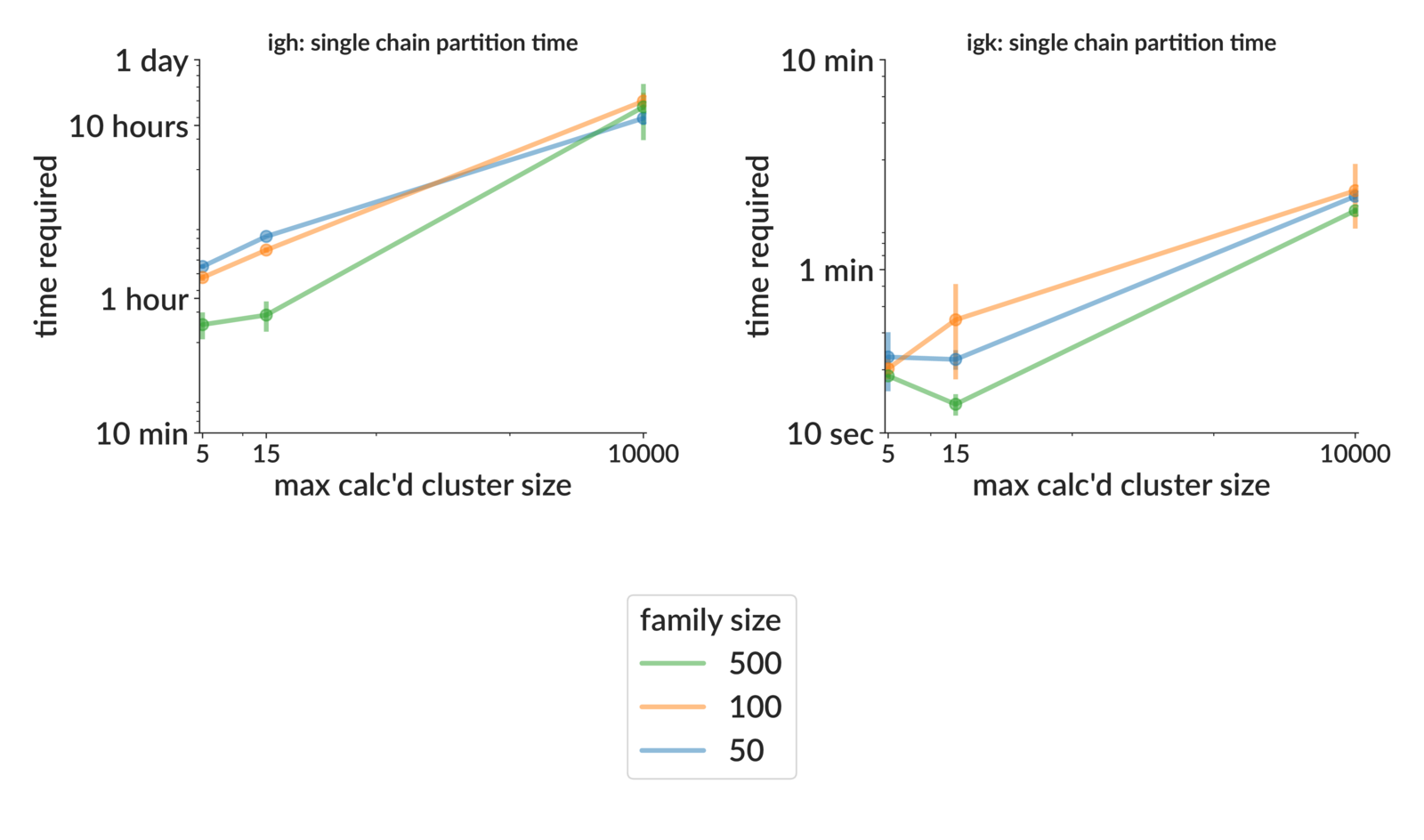

Supplement: S12 Fig — Clusters larger than (approximately) the indicated size are subsampled for the Viterbi and forward calculations during clustering (see text). (TIFF) [file pcbi.1010723.s012.tiff]

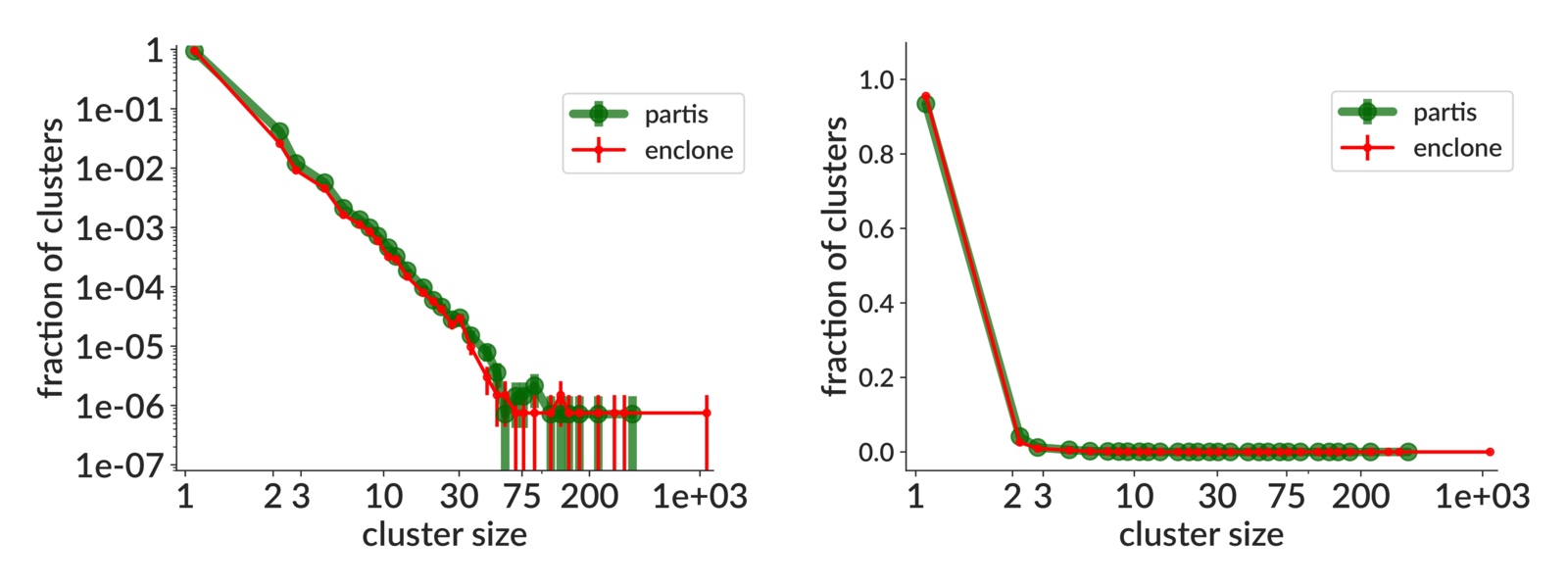

Supplement: S13 Fig — While the overall distributions are similar, enclone’s largest clusters are significantly larger. Because the “within-donor merges” column effectively squares the cluster size, these largest clusters dominate the in Table 1 of [19], which is why this number is much larger for enclone than partis (2.2 vs 1.6 million), despite partis having larger clusters for much of the distribution (as can be seen in the linear y plot, enclone has ≃2% more singletons, which is why with log y the partis line can be seen to be higher for most of the middle of the distribution). Most large enclone clusters can be constructed by merging several smaller partis clusters, then splitting off some fraction of singletons (https://doi.org/10.5281/zenodo.5860143); these two dynamics explain why enclone has many more within-donor merges (which depend almost entirely on the largest few clusters), and likely also why enclone has relatively poor sensitivity in our simulation tests (since many singletons are split from their correct family). (TIFF) [file pcbi.1010723.s013.tiff]
