# Supplementary material for: Inference of B cell clonal families using heavy/light chain pairing information
Source: PLoS Comput Biol. 2022 Nov 28;18(11):e1010723. doi: 10.1371/journal.pcbi.1010723 (PMC9731466; doi:10.1371/journal.pcbi.1010723)
Supplement: Attachment — Submitted filename: response.pdf [file pcbi.1010723.s014.pdf]

Editor

> As you can see from the review reports, all referees agreed that you present a very valuable contribution to the field of B cell clonal inference. Referee #3 raised a few important points that I would very much encourage you to address. In particular, they raised issues relating to the incompleteness discussion of previous related methodological work, the way the method has been benchmarked, and the simulated data that have been generated for benchmarking. Additionally, they remarked that data could be shared together with a more detailed description of how they were generated, either in the form of code or a more comprehensive describing text.

We thank both the editor and reviewers for taking the time to review our manuscript, and for their valuable suggestions. We are glad for the opportunity to add several substantial improvements to our analysis, as well as to clarify some important points in the text. We've written an overview here, and go into more detail inline below.

In addition to substantial manuscript revisions, we have added:

1) citations to all of the suggested papers

2) a comparison to the enclone method

3) a simulation methods section

4) a run script with instructions for reproducing all analyses.

Reviewer's Responses to Questions

> Reviewer #1: In this paper, Ralph and Matsen study the very important question of Inference of B cell clonal families using heavy/light chain pairing information. Given the increase of available single-cell data, clonal family inference based on paired chain data is an important topic that has remained underrepresented so far in the literature. The authors present a very detailed study of this question. My questions mostly pertain to the clarity of the text.

Thank you for the kind words.

> - the text is extremely detailed, which is great. However, given its extensive nature, it's at times hard to read. Can you try to shorten and make it more concise (intro and results mostly).

This is a great suggestion, we've moved the "performance metrics" section of the Results to the Methods (replacing with a short paragraph). We're having a bit more trouble finding parts of the Introduction to remove or move, but are certainly open to specific suggestions.

> - In order to be able to perform paired-chain clonal family clustering, a few preprocessing steps are required as outlined by you. Can you make a general diagram wherein you detail all the steps that contribute to paired chain clonal inference (e.g., including data cleaning, and how bulk-single cell matching can contribute to VH-VL clonal family constructions etc)?

Great idea, we have added a workflow diagram as a new Fig 2.

> - I did not find a mention of how the simulated data was generated. If it's not in the text yet, please add a description in the methods section.

Whoops, thanks. Just before submission we accidentally removed the entire simulation section! We have reinstated it, as well as expanding the bits that previously only referred to our original 2016 paper. This appears in a new subsection in the Methods section entitled "Simulation framework".

> - lines 179 – 189 don't seem to really reflect the title. Can you align these?

We're not quite sure the discrepancy you're referring to -- is perhaps the suggestion to include something about pair info cleaning and approximate bulk pairing in the title? If so, we are open to the possibility, but are inclined to say that making the title longer would not be great for clarity, and that those two tasks are mostly sub-tasks of paired clustering.

> - can you better define what you mean by partition? Also, you use the word partition a lot…so it should also be in figure 1?

Great point, we have added the following definition of "partition":

```
 We refer to the resulting clusters as a ``partition'' of the sample, and also use the verb
``partitioning'' as an alternate term for ``clustering'' that, while entirely
equivalent, emphasizes separating unrelated sequences rather than grouping related ones.
```

The figure 1 caption does refer to "partitions", although we're inclined to leave the definition only in the main text.

> - Can you make a figure for the performance metrics (purity etc)..will make it easier to understand (probably just expand Figure S2 a bit?)?

Good idea, we've added examples to S2 Figure and a mention in the text.

> - For the Bulk and single cell data augmentation investigation —> which data was used for that?

This analysis was only performed on simulation; we've added a sentence in the discussion emphasizing this:

```
 Our approximate bulk pairing method would also benefit from evaluation on real
data samples; here we have only tested it on simulation.
```

> - Can you add a datasets section to the methods section if not done already wherein you describe the experimental data used.

Good idea, we've added this information as a new "real datasets" section.

> - Do the conclusions you get on single cell data hold across different single-cell datasets? Are they dependent on B cell type (memory, plasma, naive)?

Our results are similar on the four example datasets from the 10x website that are in this paper, and on 5-10 real data samples from collaborators. We have so far run on 4 samples where we could get B cell type from gene expression data, and results seemed fine, although it's hard to know how they would look different if something were off. For instance, as we expected since there was an active infection, most expanded families were plasmablasts, and it's hard to think of a mechanism that would connect B cell type to clustering method performance, but also hard to prove that results are correct.

> - You show that family size is an important factor for obtaining correct pairing information. What's the distribution of family sizes within a given repertoire (how stable is this distribution across samples) —> based on this distribution and the accuracy values per family size, which what confidence?/accuracy can we reconstruct pairing in a given repertoire (in figure S9 you show that family size in repertoires is mostly low…which means accuracy of VH-VL reconstruction will also be low?)?

Cluster size distributions in data are essentially always rapidly and monotonically falling, with widely varying levels of clonality (Fig 7 and S9 show some examples). One useful way to characterize the clonality is the fraction of singletons, which is typically between 40% and 90%. We initially showed performance as an overall number on particular samples, i.e. with particular cluster size distributions, but decided that this is not very informative. Partly this is because the overall number depends on the particular cluster size distributions we choose, but mostly because it obscures the very simple behavior of our method's performance: it's almost always right on clusters larger than, say 5, mostly right on clusters of size 2-3, and usually leaves singletons unpaired. This behavior doesn't depend on the other clusters in the sample, i.e. throwing in a ton of singletons doesn't mess up the performance on larger clusters (it just leaves the singletons unpaired). Thus to estimate performance on a particular sample you can just take a weighted average of your preferred cluster size distribution: taking, say, 5 mean cells per droplet, a sample with 90% singletons and 10% clusters with size 10 will have around 0.2*90% of sequences left unpaired, and 0.98*10% correctly paired, with a few percent mispaired.

The key point is that, regardless of family size, mispaired sequences are rare (a few percent); thus from a user perspective, it's most useful to know that on any particular sample the singletons, and some of the doublets and triplets, will remain unpaired, but everything larger will be pretty good. We have modified the Discussion to emphasize this:

```
 This method can salvage pair info for around 90\% of sequences in families larger
than three even in simulation samples with 5-10 cells per droplet; families smaller
than this are more frequently left unpaired (especially singletons), while typically
a few percent of sequences are mispaired.
```

> Reviewer #2: This manuscript by Ralph and Matsen reports a new approach for combining information from immunoglobulin heavy and light chains to refine the accuracy of B cell clonal assignments. As clonal assignment is a critical prerequisite for many downstream analyses, improvements are always a welcome advance. Moreover, the technique is timely, as well, with the rapidly increasing adoption of single-cell sequencing techniques. In addition, Ralph and Matsen describe potentially impactful algorithms for "pair cleaning" of droplets containing multiple cells and combining single-cell (paired) with bulk (unpaired) sequencing data. I suspect that in the long run, these may prove even more useful than the refinement to clonal assignment. Overall, this manuscript represents a solid piece of work that will help the field continue to advance.

Thank you.

> Minor comments: 1. Line 63: LAIR1 insertion is thought to be an SHM-mediated event, not a part of V(D)J recombination.

> Evidence in the cited papers includes LAIR1 insertions in the switch region that are spliced into the mature mRNA between the variable and constant regions.

Whoops, thank you for the correction. We've removed the reference.

> 2. Line 102: Is this the intended reference here? Given that the sentence is about the number of cells, rather than the diversity of receptors, better references might be https://doi.org/10.1111/j.1365-2249.2010.04206.x and https://doi.org/10.1002/cyto.b.20547.

Thanks, we've added these refs.

> 3. Line 111: “0.9” needs to be labeled as referring to sequence identity (as in lines 260-261).

Thanks, fixed.

> 4. Line 127: I would add something like “have been *typically* sequenced,” to account for occasional methodological contortions as in refs 8 and 19.

Great, fixed.

> 5. Lines 146-149: “mature collisions” are not clearly illustrated in S2 Figure; I assume the intention is the two middle red circles, but this could be highlighted better.

Good idea, we've added explicit examples of naive and mature collisions.

> 6. Fig. 1: It's a little confusing that only the two a's are labeled “droplet id”. Perhaps this annotation could be left only to the figure caption, or at any rate pointed toward discordant droplet ids.

OK, moved droplet id to caption.

> 7. Line 289: I believe this should refer to S2 Figure (instead of S1).

Yes, fixed.

> 8. Lines 300-302: This sentence should be rewritten (and perhaps expanded slightly) for clarity.

We've rewritten it to:

```
 As described above, besides \shm\ fraction, clustering difficulty depends largely
on the naive collision fraction, which is in turn determined by the locus's inherent
diversity and by the number of rearrangements.
Since locus diversity is nearly constant for a given species, we next show performance
only as a function of the number of rearrangement events (families,~\ref{FIGvsNSimEvts}
and~\ref{FIGvsNSimEvtsSingleVsJointPartis}).
```

> 9. Lines 308-309: 10x doublet frequencies depend intimately on loading levels; 0.8% is the rate when 1,000 cells are loaded. At more typical loading levels, the company estimates 3-6% multiplets. See eg https://kb.10xgenomics.com/hc/en-us/articles/360059124751-Why-is-the-multiplet-rate-different-for-the-Next-GEM-Single-Cell-3-LT-v3-1-assay-compared-to-other-single-cell-applications-; even though that's 3' instead of 5', I think they are probably similar. Of course, not all of these will appear in the sequencing data as having ambiguous pairing, but I still think it's important set the scope properly.

Thanks, we've changed the number to 1-8% (to span the whole table of loading levels they give), and added a citation to that webpage.

> 10. Fig 3: Why are these simulations done with 3,000 rearrangement events instead of 10,000? Also, is 12,000 the total number of heavy+light chains, or is it 12,000 each?

The 10x data samples we've encountered range from roughly 1,000-10,000 sequences, and we wanted to mimic the upper end of that. 12,000 is the total heavy+light. We'd be happy to phrase it more clearly given a specific suggestion, but the language seems pretty close to what you're using here: "around 12,000 total heavy and light sequences"?

> 11. Lines 319-322: What happens if the number of cells per droplet is modeled (more accurately) as a Poisson distribution, instead of a uniform number? I assume this will actually result in better performance, but the authors may have better intuition here.

That's what the first version of this figure used (we switched because, as with family size, we wanted to show how performance varied in more granular detail), but, yeah, it doesn't make a significant difference -- performance basically just depends on the

mean cells per droplet. The intuition is probably that in switching from constant cells per droplet to Poisson, we do poorly on the droplets with tons of cells, but really well on those with few, and the two effects even out (not that it's obvious that they would -- that's why we checked both).

> 12. Lines 324: Many single-cell antibody experiments pre-enrich the input B cells for reactivity with an antigen of interest, which will presumably have a large effect on the family size distribution. I doubt this will substantially impact the results here, but worthwhile for the authors to note.

Good point, we've added a note to that effect:

```
 However in many practical cases, we are most interested in larger families since they
are often more likely to be responding to the antigen of interest (for instance a
vaccination dose).
Thus many experiments that pre-enrich for antigen binding will be biased toward larger
families, on which our method performs better.
```

> 13. Lines 328-330: The description of S7 Figure here is impeded by the unfortunate placement of Fig 4 immediately after – it took me a couple of reads to realize why the text and image didn't match.

Good point, we've moved Fig 4 (well now it's Fig 5) to be later, but in any case final pagination will be determined by PLOS.

> 14. Fig 4: Is the right panel calculated mutually between heavy and light, or separately and then added together? I ask because a scenario in which the similar-family rate is significantly higher for heavy chains than light chains seems plausible to me and should be explored/addressed by the authors. This might be especially important for comparing results with ref 32. A similar question might also apply to the left panel, as well as the top right panel of Fig. 3, though I am less concerned in those cases.

These are averaged over every individual heavy and light sequence, i.e. "separately and then added together". Doing it mutually would be nice, but would require ~squaring the number of categories to account, e.g. for an unpaired heavy and mispaired light chain, which is easy to code up, but would require too many plots to present. We do not report separate heavy and light numbers, since to us the most important question seems to be "what can we expect to happen to any initial [true but unknown] heavy/light pair", which since it has one heavy and one light sequence is equivalent to the values averaged over all heavy and light sequences. This is a great point though, and important to emphasize, so we've added the following:

```
 Note also that these fractions are calculated for each individual heavy and light
sequence and then averaged.
This is designed to represent the ``typical'' experience of an initial heavy/light
sequence pair, but note that each of these fractions can on average be different
for heavy chains vs.\ light chains.
This latter point means, for instance, that these numbers cannot be directly compared
to~\cite{Zhou2019-wz}, which calculates them for one chain only.
```

> 15. Lines 350-354: I was still thinking of S8 Figure when I read these lines and was confused by the apparent contradiction between the text and the bottom left panel. Perhaps another panel could be added to S8 that explicitly shows what is being described here, so as to prevent confusion.

Unless we're misunderstanding, the panel that explicitly describes this bit of text is the left panel of Fig 4 (as above, this is now Fig 5). But we agree that interspersing the descriptions of Fig 4 and S8 Figure leads to confusion between "correct family" and "correct pairing", so we've moved the reference to S8 Figure to be after the description of Fig 4:

```
 We also show the fraction correctly paired (i.e.\ paired with exactly the correct
sequence), mispaired, and unpaired (\ref{FIGpairCleanBulkDataOthers}).
```

> 16. Fig 5: Should the bottom panel include a line for paired SCOPer?

Yes good point, we've switched the paired time plot (bottom) to include single chain clustering, so we can now (and do) include methods (SCOPer and enclone) that don't separate the two.

> 17. Lines 369-370 (and elsewhere): Please don't forget to update this DOI.

Whoops, thank you. We used the version-agnostic (always-latest) link from Zenodo, but accidentally copy/pasted it after it had

redirected. Fixed now.

> 18. Lines 443-446: Something is wrong here, though I'm not sure exactly what. Perhaps "inconsistent" at the end of line 443 should be "consistent"?

Arg, thank you. We've changed "83-97%" to "3-17%" since it fits better with the "inconsistent" discussion in preceding sentences.

> 19. Lines 445-446: Isn't the more relevant comparison between the solid red line in the top left vs the one in the bottom left? Either way, I'd like to see an additional supplemental figure parallel to S4, but with [family size] on the x-axis instead of SHM.

NOTE: we assume "family size" is a typo, and think it likely should be "N families".

Here we're intending to take the "3-17% inconsistent" number as a correct estimate (i.e. neglecting effects of imperfect clustering method) of the collision rate, so we compare to the difference between clustering-method-agnostic "synthetic" grey dashed line and 1. The difference between solid red in top vs bottom left (partis single vs paired) admittedly gives essentially the same answer (taking into account the exact value of the 3% threshold in grey dashed is pretty arbitrary). But we think, philosophically, comparing single vs paired partis would be telling us how much of that collision frequency partis can recover, whereas we just want the full collision frequency.

In any case, we agree on the latter point, and have added S7 Figure comparing single vs paired partis vs N families.

> 20. Line 636: I don't think I am understanding what it means to "remove both sequences." In Fig. 10, both droplets are shown as having 3 "k1" pairings, suggesting it hasn't been removed after the first one was disambiguated.

There's two different places from which they could potentially be removed: the list of potential partner sequences, and the counting info on how many votes there are for each partner family. We remove them from the former (since we've decided who they're paired with, we don't want to pair them with anyone else), but not the latter (since that would gradually destroy all the information we have about who pairs with whom; which, incidentally, we figured out by accidentally implementing it that way originally). We're not sure that there's a good way to convey this in the cartoon, so are inclined to leave it as is, but we've added the following parenthetical statement:

```
 Whenever we choose a unique partner for a sequence, we remove both sequences from
the list of potential partners for all other sequences.
```

> 21. References 16, 21, 33, and 40: Journal name is corrupted/missing.

Fixed, thanks.

> 22. S8 Figure: there seems to be an issue with the way lines at zero are displayed in the bottom two panels.

Fixed, thanks.

> Reviewer #3: Ralph & Matsen describe an extension of their previous work on single immunoglobulin chain B cell clonal inference into the setting of paired chains, and benchmark their method against some existing ones. They also describe an algorithm that attempts to tease out the "true" immunoglobulin pair in a cell that happens to contain more chains than expected. In addition, they describe a procedure that matches an unpaired chain approximately to a paired BCR. Overall, paired chain clonal inference is a welcoming addition to the field, especially in light of the ever increasing volume of single-cell paired BCR data, along with continued efforts of bulk BCR-sequencing. Several issues of concern are noted below, mainly the omissions of relevant developments, the inclusions of which for comparison would be highly informative. On the other hand, I am of the opinion that there is very little novelty about the "approximate bulk pairing" procedure.
>
> The authors do a commendable job in the Introduction covering a wide range of immunological backgrounds relevant to the task of clonal inference, albeit with several scientifically important omissions and inaccuracies, some of which have implications for their subsequent benchmarking work and beyond.
>
> First, the authors claim that the widespread practice when it comes to clonal clustering is to do so based on CDR3 sequence with a threshold that is "typically 0.9 [11]" (L111). Aside from the fact that there is no mentioning whether this is a threshold for nucleotide sequences or amino acid sequences, it is highly questionable whether 0.9 is a "typical" threshold. The reference given, [11], is not a review article; nor is it an acceptable reference to support this claim. I could not find any discussion regarding clustering threshold in [11]. If anything, [11] speaks against 0.9 being typical as [11] appears to be using a threshold of 1 -- "Clonotypes were identified on the basis of ... identical amino acid sequences of the heavy-chain CDR3".

Apologies, this was the incorrect reference. The reviewer makes a great point that "typically" is too strong a word, and there isn't a real reason to have a threshold example listed at this point in the paper, so we've removed it. We thus defer discussion of specific thresholds to line 262 (of the submitted version), where the actual method is described, and which when submitted read: "we use a threshold of 0.9 in nucleotide distance and call it VJ CDR3 0.9".

> It has been my observation that many works in the field tend to use a threshold -- nucleotide or aa -- that is lower than 0.9. Examples abound, but just to list a few: Galson et al. use 0.8 (nucleotide); Vander Heiden et al. use 0.75 (nucleotide); Nielsen et al. use 0.85 (aa). On the other hand, Soto et al. and Briney et al. both use 1 (aa). In addition, by claiming that 0.9 is "typical" and proceeding to use for some of the benchmarking such a high threshold that many in the field do not use, the authors risk artificially deflating the sensitivity performance of the "VJ CDR3" method.

We are not attached to any particular threshold value, and have thus switched to one of the two values suggested by the reviewer (0.8, from the more recent paper). This does not materially affect the results.

> Second, the coverage on current clonal inference methods is far from being complete. Given that the authors note in Discussion that their paired clustering could likely improve if they "used pairing information from the start" (L457), an important fact missing from their survey is that there have already been works that use paired heavy and light chains "from the start" for clonal inference. Some examples include Soto et al., Alsoussi et al., and Kim et al., all of which use heavy and light chains simultaneously when partitioning paired BCRs -- as opposed to partitioning by heavy first, then by light chains, which is what the SCOPer method benchmarked does. One-step partitioning could be of interest for benchmarking as well, in addition to the benchmarked two-step partitioning.

Thank you for pointing out these references. The focus of our paper was to benchmark against existing implementations that were available as software packages. The reviewer is right that these are important papers to cite, however the lack of an implementation means that we cannot make a direct comparison. We have added the following:

```
 Some previous experimental papers have used various methods to cluster paired data,
either by hand or with non-public code.
For instance~\cite{Soto2019-ed} grouped together sequence pairs with identical heavy
and light V and J genes, and identical CDR3 amino acid sequences.
Two other recent papers similarly grouped together identical heavy and light V and
J genes and CDR3 lengths, but then clustered with \scoper's hierarchical method on
heavy chain only~\cite{Alsoussi2020-hk,Kim2022-wg}.
```

> Given that the authors state that a fundamental issue affecting existing methods is that somatic hypermutation tends to cause biological clones to be computationally split (L115), another notable omission from Introduction is therefore the development of methods that attempt to address exactly this issue. Nouri & Kleinstein (2020), for instance, developed an approach that makes use of shared SHMs when inferring clonal relationships. This method is not cited, but should be. In fact, unless mistaken, this method is accessible via SCOPer through specifying `vj="vj"` in the very script (https://bit.ly/3K8KfBa) that the authors use on L268 (In the link, `vj` appears to have been set to "novj", meaning no SHM information is to be used). `enclone` also attempts to address the SHM issue (see below).

While the default setting in the script that the SCOPer authors sent does not use shared mutation, the command line with which they instructed us to run the script does use it: https://github.com/psathyrella/partis/blob/main/test/scoper-run.py#L98. Also, it is not true that using shared mutation always improves performance on high mutation samples. Particularly when clustering on observed (i.e. mutated, rather than naive) sequences, shared mutation will indeed help in grouping together members of a sublineage, but it also assumes that all families consist of only one sublineage, and will thus spuriously split families with several widely separated branches (this is illustrated in S2 Fig). For example in the AbSim performance table below, the SCOPer shared shm option performs generally worse than the default SCOPer.

We have added a clarification in this respect, along with a citation to Nouri & Kleinstein 2020:

```
 Some methods also group together sequences with similar inferred mutations, and split
apart those with different ones~\cite{Nouri2020-yo,Jaffe2022-enclone}.
This can improve performance for instance on trees with shared mutations on an edge
descending from the naive ancestor.
However, if there are not enough mutations shared among all sequences then this can
incorrectly split lineages apart (\ref{FIGclusterError}).
```

And we have added this clarification to the caption of S2 Figure:

```
Note that this figure also shows a potential problem with using shared mutations as
a criterion for clustering: while it can help in grouping together members of a sublineage
in a high mutation environment, it assumes that all families consist of only a single
sublineage, and will thus spuriously split families that do not (such as the five-sequence
family at bottom left above).
```

> More importantly, given that the authors claim that their previously published work on single-chain clonal inference [12], to which the current manuscript appears to be a paired-chain extension, addresses the abovementioned SHM issue (L120), it seems to me that the current setup of their benchmarking -- partis vs. a variant of SCOPer (spectral clustering, single chain or paired) that doesn't use SHM information -- is unfair, as it compares a method designed to overcome the SHM issue, against a method for which the SHM issue is a known problem. A fairer comparison, in my opinion, would thus be to compare partis against the variant of spectral clustering that does take into account of shared SHMs, i.e. the method proposed by Nouri & Kleinstein (2020) and implemented in SCOPer.

We suspect that the reviewer means "shared SHM", as all SCOPer methods take into account SHM; a method that did not would be a TCR clustering method, not a BCR one. As noted above, we think it more appropriate to use the method recommended by the SCOPer authors, which does not use shared mutations. It is perhaps worth adding that partis does not use shared mutation information in clustering, due to potential downsides of that approach.

> A related note regarding benchmarking is the seeming discrepancy in the performance of some of the methods benchmarked here, compared to elsewhere. For instance, one of the methods that the authors benchmark partis against, spectral clustering without using SHM information [17], is reported to have median sensitivity in the range of 96%-98% when benchmarked in the original publication [Fig 9 of ref. 17; specificity was reported instead of precision] against repertoire data simulated by Ralph & Matsen, 2016 [12] with 0.10 fraction of SHM. In Fig S5, however, the authors report that single chain scoper [17] has "igh: sensitivity" around 0.9 at SHM level of 0.10. The magnitude of performance discrepancy of the same method in two separate benchmarking settings brings the question as to how much of the better performance by partis and worse performance by [17] can be attributed to the inherent differences in the simulated repertoire data used for benchmarking. In other words, is the seemingly improved performance of partis reported here going to hold when applied on the simulated data used in [17], or, as suggested earlier in the review, the mutated repertoire data simulated using AbSim used in Fig 5 of Nouri & Kleinstein (2020) if the benchmarking were to be performed against spectral clustering using shared SHM information? After all, there is little to no description in the current manuscript as to how the authors generated their simulated datasets this time. It thus seems not only fair but necessary to perform benchmarking on not only the authors' own simulated data, but also the alternative methods' original simulated data.

There is no single definition of precision or sensitivity (or specificity or recall) in clustering performance: there are many different ways to calculate two complementary numbers quantifying over- and underclustering. Each paper typically chooses one method, to which are then applied the familiar nomenclature of classification performance. The indicated paper (Nouri 2018) uses a definition:

[...] the sensitivity of the method is defined as the fraction of all sequence pairs from the same clone that were correctly inferred by the method, while specificity is defined as the fraction of pairs of unrelated sequences that were successfully inferred by the method to be in different clones.

That is very different to ours:

```
While there are several different ways to calculate these numbers~\cite{Abdollahi2021-qx},
we calculate precision as the fraction of sequences in a cluster that are truly clonal
(\ref{FIGclusterError}), and sensitivity as the fraction of a true clonal family that
end up together, each averaged over all sequences.
```

The indicated samples also have many parameters different from those in our current paper, e.g. family size distributions, germline sets, and gene probabilities. As described in the text, we have made a real effort to match these to real data. In addition, it is unknown to what extent the spectral SCOPer method has been updated in the intervening four years, or if the author's recommended commands for the current paper are the same as those used in the original 2018 paper.

Regarding running validations on the other simulations, we agree that this is a good objective, however, the suggested simulation frameworks appear to only support single chain simulation. Specifically, [17] only considers heavy chain, while the documentation for AbSim lists only an option to choose heavy or light chain alone, but nothing to implement the coordination necessary for paired simulation. If we have misunderstood opportunities to benchmark paired simulation using these previous methods, please let us know and we will add it.

However, just out of curiosity we have run SCOPer with and without shared shm, as well as partis, on four of the AbSim samples from the indicated paper. Interestingly, SCOPer seems to perform better on these samples without shared mutation, and partis appears to perform better than either SCOPer version (the pair of numbers in each method's column are precision, sensitivity):

| sample | shared scoper | non-shared scoper | partis | mean % shm |
|---|---|---|---|---|
| 1 | 0.73, 0.91 | 0.75, 0.92 | 0.98, 0.96 | 8.5 |
| 2 | 0.89, 0.85 | 0.96, 0.86 | 0.99, 0.92 | 9 |
| 3 | 0.91, 0.95 | 0.93, 0.95 | 0.98, 0.98 | 5 |
| 18 | 0.97, 0.93 | 0.98, 0.93 | 0.98, 0.99 | 4 |

> Another major omission from existing clonal clustering methods that would be particularly relevant for benchmarking is `enclone` from 10x Genomics (https://support.10xgenomics.com/single-cell-vdj/software/pipelines/latest/algorithms/clonotyping/), especially given the current widespread adoption of 10x Genomics' products for single-cell paired BCR profiling, as well as the fact that `enclone` is built into `cellranger`, meaning that anyone processing 10x Genomics data with `cellranger` would have access to clonal inference results produced by `enclone` (whether they use those results is a separate question). Granted, a formal `enclone` preprint was only recently posted by Jaffe et al.; however, `enclone` itself has been in production since cellranger 5.0.0 as of 2020-11-19. Of note, `enclone` utilizes both heavy and light chains, as well as shared SHM when inferring clones, making it a particularly useful candidate for benchmarking. In addition, while not exactly the same as the "pair info cleaning" that authors propose here, `enclone` also handles the presence of multiple immunoglobulin chains.

Great suggestion, we saw that the enclone preprint was posted while we were waiting for these reviews, and we've added enclone to the performance comparisons. As far as is described in the preprint and online documentation, enclone's handling of multiple heavy or light chains consists of a series of heuristics that attempt to discard extra sequences in doublets (droplets with multiple cells), which is a different goal than pair info cleaning.

> On disambiguating multiple pairing information, the authors claim that cells with more-than-expected number of chains are "typically discarded [36]" (L311). Several recent studies, in contrast, strike a middle ground between straight out discarding and preserving everything. For instance, Jiang et al. perform "pair info cleaning" before clonal inference by preserving the most abundantly sequenced heavy chain as indicated by UMI count. Lee et al., taking a different approach, keep the lambda chain and discard the kappa chain when both are present, reasoning that lambda rearrangement only happens if kappa failed. Both approaches are not without biological basis, and should be referenced. Benchmarking comparison would be of interest if at all possible. In particular, in cases where both kappa and lambda chains are present in a cell, should the vote-by-majority approach suggested by the authors (L632) be in favour of keeping the kappa chain, it seems not unreasonable to say that Lee et al.'s approach would produce a result more in line with the actual underlying B cell development.

It is a great point that "typically" is too strong -- we have changed it to "often".

These are great studies to reference, thanks for pointing them out. Since they address the various ways that we can find multiple chains stemming from a single cell, however, they focus on a different problem to that which we tackle in this paper: multiple cells per droplet. We have thus added the following:

```
Prior work in the vein of our pair info cleaning, as far as we are aware, consists
of custom approaches applied in experimentally-driven papers aimed at addressing
multiple chains per cell (i.e.\ allelic inclusion).
In this paper, on the other hand, we focus on creating a method for handling multiple
cells per droplet (although in future versions we plan to address multiple chains
per cell).
In~\cite{Jiang2020-ju}, for instance, only the heavy chain sequence with the most
unique molecular identifiers (UMIs) is retained from any droplets with multiple
heavy chains.
In~\cite{Lee2021-ip}, on the other hand, IgK reads are discarded from any droplets
that have both IgK and IgL, since if both are from the same cell, the IgL will only
have rearranged upon failure of IgK.

It is also possible to use experimental techniques to allow droplet overloading,
for instance by pooling samples with distinguishable genotypes~\cite{Kang2018-iq}, or
by adding sample-specific barcodes to surface-protein-binding antibodies~\cite{Stoeckius2017-tx}.
We note, however, that without a computational disambiguation scheme, these methods
would likely still be limited to only one cell per droplet from each subject.
```

> I find the descriptions of the results of validating simulation against real data to involve too much hand waving. The authors describe the clone size distributions from simulated data and real data (Fig 6) as sufficiently similar to provide confident validation (L373-374). Looking at Fig 6, the actual level of similarity seems to be a rather subjective matter, especially when compared to S9 Figure. In the latter, there is little doubt that the curves for real and simulated data match ever so closely. In Fig 6, on the other hand, the differences are quite pronounced. It would be informative to quantify the comparison of distributions using a metric. More importantly, to convincingly demonstrate that the simulation, with the parameters used, reasonably and sufficiently matches real data, it would be important to have some form of "negative control" -- for example, simulated data generated with parameters that are wildly different from what's expected of reality.

In the text we do not say that the level of agreement "provide[s] confident validation", we say that "they give us confidence that [the simulations] suitably approximate the underlying processes for the purposes of benchmarking". As we explain there, we do not expect the distributions in Fig 6 to match exactly, since we do not model biological pairing correlation (which we know is present at some level), and because the data sample that we are mimicking is extremely small. Furthermore, biological pairing correlation would shift the curves exactly as we observe -- we would see more simultaneous heavy and light collisions, and thus less difference between the single and joint partitions. The distributions in S9 Figure, on the other hand, compare true simulation parameters to the inferred data distributions they are mimicking, and thus their agreement does not depend on either inference or (through it) biological pairing correlation. We agree that it would be preferable to quantify the level of expected discrepancy, however this is not possible without measuring biological pairing correlation, which we intend to do, but is not within the scope of this paper. We also note that the choice of log-log axes, while necessary to display these comparisons, is likely over emphasizing relatively minor discrepancies, while obscuring the general agreement of two orders of magnitude on the x axis and almost four orders of magnitude on the y axis. Stepping back a little, what we want to do is to set up a simulation which tests the paired part of this inferential procedure: how hard does the algorithm have to work in order to reconcile differences between the single-chain clustering and paired clustering? From this perspective, we note that the difference between data and simulation is small relative to the difference between the single chain and joint clustering. We have thus added the following to clarify this point:

```
It is possible that this reflects a breakdown of the assumption of no biological
pairing correlation: low (but non-zero) correlation in data would manifest as having
split fewer than expected light clusters based on heavy information (and vice versa).
Overall, because these discrepancies are much smaller than the difference between
the light chain single and joint distributions, which control the difficulty of
inference, we think this level of agreement is sufficient for our current benchmarking
purposes.
In the future we plan to investigate biological pairing correlation in data, at
which point we will be able to more thoroughly investigate this.
```

Regarding a "negative control" we don't follow how this would bring clarity: we are trying to show that our simulation framework and parameter values are accurate enough for the purposes for which we use them, not that other frameworks or parameter

values would be problematic.

> In general, there is very little detail on how simulations were performed, both for paired chain clonal inference, and for pair info cleaning. This makes it hard to evaluate whether the simulated datasets are appropriate, and impossible for future readers to replicate such simulations. The Zenodo link provided that is supposed to contain simulated data appears to be a simple data dump with no metadata description whatsoever, and as such is not at all helpful in this regard.

Yes, thank you -- as we note above (Reviewer #1), we inadvertently deleted the simulation methods section just before submission. We have reinstated and expanded it. We also apparently forgot to include a link to the run script in the paper, so we apologize, also for that oversight. We've thus added the following:

```
 All inputs and outputs can be found at~\zenodolink; however any part of the analysis
can also be rerun, for instance with modified parameters, using~\runscript.
```

We have also added a README to the Zenodo link.

> The authors try to match bulk-sequenced unpaired chain with single-cell paired chains using clonal family information. They call this "approximate bulk pairing", emphasizing that the unpaired chain can be matched with an opposite chain from the correct clonal family, but not necessarily the correct sequence. They argue that this is in practice "sufficient to create a functional antibody" (L664-671). In essence, their algorithm matches an unpaired chain with the pair of chains containing the unpaired chain's nearest neighbour in the sequence space. While the preparatory work leading up to this matching step -- that is, single chain partitioning and paired chain partitioning described in earlier sections -- is novel, this matching algorithm itself is not novel (L454). The practice of using single-cell paired chains as anchor to "fish" amongst bulk unpaired chains has been used both in academia and in industry for antibody discovery. Multiple COVID-19 works use this strategy. Kim et al., for instance, paired bulk-sequenced bone marrow heavy chains with light chains from single-cell plasmablasts with paired chains from the same single chain partition, expressed the antibodies, and tested for neutralization.

This is a great point, we have added the following reference:

```
 There have also been experimental papers that use individual families in single
sequence data to look for similar sequences in a matched bulk sample, for instance~\cite{Kim2022-wg}.
This addresses the same need as our approximate bulk pairing, however our method
provides a codified, repeatable method together with validation.
Our method also operates comprehensively on the entire repertoire at once rather
than focusing on a single target sequence at a time.
```

> --- Minor --- L180-181: "as well as a paired method that has been used [34]" While Hoehn et al. [34] did use said method in their 2021 work, the same method was first used in 2019 by Oh et al., which would be the correct reference instead. From Oh et al.: "In addition, Igh V(D)J sequences were further grouped based on whether the associated cells shared common combinations of Igk V with Igk J or Igl V with Igl J gene annotations." This step was done via the `light_cluster.py` script from Immcantation, which [34] also used.

Apologies -- we used the reference to which the SCOPer authors directed us. We have added this reference as well.

> Fig 2 caption: "All methods are publicly available except for SCOPer, whose paired method was obtained via personal communication." It is factually incorrect to say that the SCOPer paired method is not publicly available. The script "obtained via personal communication", referenced on L268 at https://bit.ly/3K8KfBa, is nothing more than a wrapper making calls to functions all of which are available on CRAN. In fact, to use the paired method, one need not use the script at all. As long as one specifies `only_heavy=FALSE` to either `hierarchicalClones` or `spectralClones`, both functions from the publicly available SCOPer R package, one could use the paired method. Just like a user would have to specify different parameters to use partis, the program written by the authors, to perform a task -- such as clonal clustering using paired information as covered in this manuscript, having to specify parameters to use SCOPer does not make it publicly unavailable.

Apologies -- we tried several different ways to describe the situation when we initially wrote the first draft of this paper, which was that the SCOPer authors mentioned the existence of a paired method in the online documentation, but not with enough detail to use it, nor with any reference describing how it worked. This situation has since been remedied with much more detailed documentation, which is greatly appreciated. We eventually settled on the "not publicly benchmarked" language in the introduction, but missed updating this figure caption. We've removed the offending sentence.

> Fig 4, left vs. S8 Figure, top: These appear to show results for similar, if not identical, benchmarking comparisons. However, the patterns and numerical range are completely different for all the curves except for the one corresponding bulk data frac = 0. What is going on?

Fig 4 is the fraction paired with any sequence from the correct family, S8 Figure is with the actual correct sequence, the latter of which, by construction, is just the bulk data fraction. The captions read: "shown as the fraction of sequences paired with a sequence from the correct family (but not necessarily the correct sequence, left)" vs "sequences correctly paired (top)", although we're open to suggestions for clarification.

> L296-298 "a lot", "a little" -- More rigourous and quantitative descriptions should be used instead of casual colloquialism.

We have just dropped these, thanks.

> L300-302: "we show performance as a function of the distance between naive rearrangements only versus the number of rearrangement events (S6 Figure)." I could not find a S6 Figure matching the text description on L300-302. The S6 Figure present shows "Clustering performance on simulation as a function of the number of families". Could there have been a mix-up?

A clonal family is the result of a rearrangement event, so these are the same. We've added a parenthetical "families" to the text to clarify.

> L215 "they perfectly recapitulate the underlying biological processed" -- Is this meant to be "imperfectly"?

No, we were saying that, by definition, real data perfectly recapitulates the actual biological process. We've modified the sentence to say that real data "embody" the underlying process, which hopefully is clearer.

> L470-472: "We also note that we have used the pair cleaning algorithm with good results on (not-yet-published) non-10X data that flow-sorted cells into wells, but left multiple cells in some wells. "It is of course up to the editor to decide, but I do not find an unsubstantiated statement such as this one that is essentially "data not shown" based on "not-yet-published" data to be acceptable for peer-reviewed publication.

We are also happy to defer to the editor on this. We think it would be useful for readers to be aware of this additional situation in which our methods could be useful, so if the mention of unpublished data is unacceptable, we could instead (misleadingly) say that we could imagine the method could be useful in this additional situation.

> Last but not least, I find the entire section of "General partis improvements", including Fig 8, to be completely out of place. Whilst partis might be an all-encompassing software that performs V(D)J annotation, clonal inference, and other tasks, I do not find it relevant or appropriate to piggyback updates concerning V(D)J annotation, a task upstream of clonal inference, into a manuscript that is supposed to be about an extension of single chain clonal inference into the paired chains setting. I suggest that this section be taken out and submitted elsewhere, for instance, perhaps as an Application Note at Bioinformatics. Again, this is, of course, up to the editor.

Every paired clustering result that we present here depends on and uses these general partis improvements, so they are certainly related to the main thrust of the paper. We agree that many readers will only be interested in the novel paired methods, however this is why we have structured the "general improvements" into separate, self-contained sections that such readers will have no trouble ignoring.


> --- References --- Galson et al. Deep Sequencing of B Cell Receptor Repertoires From COVID-19 Patients Reveals Strong Convergent Immune Signatures. Front. Immunol., 2020. Vander Heiden et al. Dysregulation of B Cell Repertoire Formation in Myasthenia Gravis Patients Revealed through Deep Sequencing. J. Immunol., 2017. Nielsen et al. Human B Cell Clonal Expansion and Convergent Antibody Responses to SARS-CoV-2. Cell Host & Microbe, 2020. Soto et al. High frequency of shared clonotypes in human B cell receptor repertoires. Nature, 2019. Briney et al. Commonality despite exceptional diversity in the baseline human antibody repertoire. Nature, 2019. Alsoussi et al. A Potently Neutralizing Antibody Protects Mice against SARS-CoV-2 Infection. J. Immunol., 2020. Kim et al. Germinal centre-driven maturation of B cell response to mRNA vaccination. Nature, 2022. Nouri & Kleinstein. Somatic hypermutation analysis for improved identification of B cell clonal families from next-generation sequencing data. PLOS Comp. Bio., 2020. Jaffe et al. enclone: precision clonotyping and analysis of immune receptors. BioRxiv, 2022. https://www.biorxiv.org/content/10.1101/2022.04.21.489084v1 Jiang et al. Single-cell repertoire tracing identifies rituximab-resistant B cells during myasthenia gravis relapses. JCI Insight, 2020. Lee et al. Long-lasting germinal center responses to a priming immunization with continuous proliferation and somatic mutation. BioRxiv, 2021. https://doi.org/10.1101/2021.12.20.473537 Oh et al. Migrant memory B cells secrete luminal antibody in the vagina. Nature, 2019.

>
> Have the authors made all data and (if applicable) computational code underlying the findings in their manuscript fully available? Reviewer #1: Yes Reviewer #2: Yes Reviewer #3: No: Simulated data is provided but without any associated metadata that makes it usable. No code and little-to-no method description in text are provided for generating the simulated data.
