# Supplementary material for: Inference of B cell clonal families using heavy/light chain pairing information
Source: PLoS Comput Biol. 2022 Nov 28;18(11):e1010723. doi: 10.1371/journal.pcbi.1010723 (PMC9731466; doi:10.1371/journal.pcbi.1010723)
Supplement: Attachment — Submitted filename: response.pdf [file pcbi.1010723.s015.pdf]

> As you will see two of the three reviewers are happy with your revisions. Reviewer #3 raises a couple of points relating to how previous work is being discussed. The first point concerns the level of validation. In the view of the editor a biological validation is beyond the scope of the study, but the reviewers criticism could be adequately addressed by calling the validation "computational validation", "validation by simulation" or similar. We also encourage you to consider providing a description of the clustering in the study by Soto et al. more nuanced than captured by the phrase "by hand".

We have changed the phrase to "computational validation", this is a great suggestion. On the second point, we cannot add much detail on that method, since the paper offers few details, but we agree that the phrase "by hand" is too vague, so we have removed it.

> Reviewer #1: The authors have addressed all my comments.
>
> Reviewer #2: The authors have done a thorough job of responding to the critiques and suggestions of all three reviewers. I have no further comments at this time.
>
> Reviewer #3: The revised submission constitutes an improved manuscript. The authors attempted to address most comments reasonably and sufficiently. Two minor points remain objectionable.
>
> The authors tried to frame as an advantage of their approximate bulk pairing method that it "provides a codified, repeatable method together with validation" [L421, version showing changes]. Soon after, nonetheless, they note that "[o]ur approximate bulk pairing method would also benefit from evaluation on real data samples; here we have only tested it on simulation" [L455-456]. First, the so-called "validation" for the approximate bulk pairing method was purely computational, based on simulation, and using one of various metrics possible for evaluation -- in this case, similarities in clone size distribution (Fig 4). Whether that is the optimal metric for benchmarking is by no means a given and a separate question. In contrast, the existing work cited, [52], carried out actual, real-world, biological validation. Antibodies that were formed by computationally pairing heavy and light chains were experimentally synthesized, functionally characterized, and confirmed to bind and neutralize SARS-CoV-2 spike. When it comes to validation of BCRs/antibodies, the only gold standards are functional assays. Otherwise, there is simply no telling whether the pairing suggested by this approximate bulk pairing method would give rise to an experimentally viable antibody that can be successfully expressed, remain stable, and bind its cognate antigen. After all, if not for binding, what's the point of having a paired BCR at all? In other words, there was no biologically meaningful "validation" here [L421] and the method indeed could benefit from actual experimental validation that is more robust than what's proposed on L455-456.

We think that adding "computational" before validation is a great suggestion to clarify our approach, and have made this change in the manuscript. However, we would also like to clarify that the reviewer is mistaken -- we do not use the clone size distribution to evaluate bulk pairing accuracy. The bulk data pairing validation figure (Fig 5 at the moment -- Fig 4 is pair info cleaning) measures performance using the fraction paired with "correct" and "similar" families, not with the clone size distribution. We agree that the clone size distribution would have been a wildly inappropriate performance metric for this section. The places we do use the clone size distribution for validation are when comparing with real data (which, as they correctly note, we do not do for approximate bulk pairing): Fig 7 (paired clustering method), Fig S10 (simulation accuracy), and Fig S13 (partis vs enclone paired clustering). If there are ways we could make this more clear we would be more than happy to do so.

It is also true that experimental synthesis is the only way to know if two sequences form a functional antibody; we've added the following sentence to emphasize this:

```
 Finally, it is important to note that the only way to be certain that an incorrect
pairing (even within the correct family) results in a functional antibody is to actually
synthesize it.
```

> In comparing with other prior works [50-52], the authors also casually suggested that these works clustered paired data "by hand" [L381]. It is understandable that when trying to promote one's own method, one would like to highlight the perk of their method over others'. But this is just silly and grossly inaccurate. [50] reported 9-17 million B cell clonotypes; I find it extremely unlikely that Soto et al. sat down and painstakingly went through millions of BCRs, sequence by sequence, "by hand", to cluster them. Clearly Soto et al. would have used some program or implemented some custom code, both scenarios already covered by "with non-public code" [381]. In short, I suggest that the inaccurate characterization of others' works, "by hand", be dropped.

We agree that "by hand" is vague enough to not be useful, so we have replaced the phrase with "non-public implementations". What we meant to suggest with the phrase was an interactive R session or an Excel spreadsheet.
